# A Method for Correcting Signal Aberrations in Ultrasonic Indoor Positioning

**DOI:** 10.3390/s24062017

**Published:** 2024-03-21

**Authors:** Riccardo Carotenuto, Demetrio Iero, Massimo Merenda

**Affiliations:** 1 Department of Information Engineering, Infrastructure and Energy Sustainable (DIIES), Mediterranea University of Reggio Calabria, 89124 Reggio Calabria, Italy; demetrio.iero@unirc.it (D.I.); massimo.merenda@unirc.it (M.M.); 2 HWA srl, Spin-off Mediterranea University of Reggio Calabria, Via R. Campi II tr. 135, 89126 Reggio Calabria, Italy

**Keywords:** acoustic signal aberration, cross-correlation aberration, ultrasonic ranging, acoustic SNR

## Abstract

The increasing focus on the development of positioning techniques reflects the growing interest in applications and services based on indoor positioning. Many applications necessitate precise indoor positioning or tracking of individuals and assets, leading to rapid growth in products based on these technologies in certain market sectors. Ultrasonic systems have already proven effective in achieving the desired positioning accuracy and refresh rates. The typical signal used in ultrasonic positioning systems for estimating the range between the target and reference points is the linear chirp. Unfortunately, it can undergo shape aberration due to the effects of acoustic diffraction when the aperture exceeds a certain limit. The extent of the aberration is influenced by the shape and size of the transducer, as well as the angle at which the transducer is observed by the receiver. This aberration also affects the shape of the cross-correlation, causing it to lose its easily detectable characteristic of a single global peak, which typically corresponds to the correct lag associated with the signal’s time of arrival. In such instances, cross-correlation techniques yield results with a significantly higher error than anticipated. In fact, the correct lag no longer corresponds to the peak of the cross-correlation. In this study, an alternative technique to global peak detection is proposed, leveraging the inherent symmetry observed in the shape of the aberrated cross-correlation. The numerical simulations, performed using the academic acoustic simulation software Field II, conducted using a typical ultrasonic chirp and ultrasonic emitter, compare the classical and the proposed range techniques in a standard office room. The analysis includes the effects of acoustical reflection in the room and of the acoustic noise at different levels of power. The results demonstrate that the proposed technique enables accurate range estimation even in the presence of severe cross-correlation shape aberrations and for signal-to-noise ratio levels common in office and room environments, even in presence of typical reflections. This allows the use of emitting transducers with a much larger aperture than that allowed by the classical cross-correlation technique. Consequently, it becomes possible to have greater acoustic power available, leading to improved signal-to-noise ratio (SNR).

## 1. Introduction

Emerging technologies like augmented reality (AR) and various positioning-based applications are driving the need for indoor positioning technology. Applications such as mall navigation, pathfinding in large hospitals or airports, automatic guidance for unmanned cleaning and maintenance vehicles, surveillance systems, and others demand positioning systems capable of delivering high accuracy within indoor environments [1,2,3,4,5,6]. Trilateration, a well-established technique, is commonly employed in accurate positioning systems for indoor use. This method relies on three (or more) range measurements between reference emitters and a sensor to determine the distances and spatial positions with a high degree of precision at a relatively low cost. Ultrasonic wave-based systems have proven effective in providing accurate positioning [7,8,9,10,11].

The widely used ranging technique involves estimating the time of arrival (TOA) of a suitable ultrasonic signal. Typically, TOA is determined by identifying a specific feature in the received signal or the post-processed signal that is easily recognizable upon arrival. In certain situations, the determination of the time of arrival (TOA) often relies on pinpointing the maximum peak within the envelope of an ultrasonic pulse. However, despite the apparent simplicity of this method, it remains susceptible to disruptions caused by acoustic interference, leading to errors on the scale of wavelengths (centimeters), even with a high signal-to-noise ratio (SNR) [11,12,13,14,15].

Methods employing cross-correlation, on the other hand, provide a markedly precise estimation of TOA and generally demonstrate robust resistance to acoustical disturbances [14,15,16,17,18]. Digital cross-correlation techniques proficiently sample and transform the received acoustical signal into the array ***C*** = ***S*** ★ ***T***. In this context, ***S*** denotes the numerical array of received signal samples, ***T*** represents the digital reference signal stored in the sensor processor memory as a numerical array of samples, and ★ serves as the cross-correlation operator. The optimum alignment of ***S*** and ***T*** in time is discerned through the peak of the cross-correlation. The displacement between signals, or lag τ, corresponding to the cross-correlation peak (i.e., τ_MAX_), is directly proportional to the TOA [19,20,21].

The precision in measuring distance within the current spatial sampling framework—defined as the distance traversed by ultrasound during the time sample interval—can be markedly reduced compared to the ultrasonic wavelength. This enhanced accuracy is achieved by determining the TOA through the identification of the cross-correlation peak.

In practical systems, a range resolution can be achieved up to approximately one-tenth of the wavelength used. For instance, in [16], an experimental range resolution of ±1.2 mm was attained using a 15–40 kHz chirp, with a wavelength range of 22.86–8.57 mm. This was realized considering a sampling frequency of 1 MHz, resulting in a space sampling of 0.34 mm, and utilizing a sound speed of 343 m/s. In cases where the signal and noise are uncorrelated, the process of cross-correlation significantly enhances the signal-to-noise ratio (SNR), commonly referred to as process gain.

Extensive research has been conducted in the past on the acoustic field produced by acoustic transducers, considering impulsive or continuous sinusoidal wave signals and varying the shape and aperture of the transducer. The exploration of closed-form solutions for the generated acoustic field has facilitated the derivation of simple approximate formulas for calculating the emission angle. These formulas depend on factors such as wavelength, aperture, and distance from the emitter, with the most recognized ones being applicable to circular apertures. In the far field, the semi-angle *ϑ* of the emission cone is approximately characterized by the well-established relationship [22]:

(1) $$\sin\vartheta =1.22\frac{\lambda }{D}$$

where *D* is the diameter or aperture of the transducer and *λ* is the emitted wavelength.

Furthermore, it is well established that achieving optimal results in signal reception requires the receiver to consistently operate within the designated “emission cone” of the emitter [23]. Presently, there are no equivalent formulas applicable to signals of varying shapes, such as chirp signals. In the development of a positioning system based on ultrasonic signals, the utilization of numerical tools becomes essential to evaluate the spatial coverage of each transducer in terms of the quality (including amplitude and deformation level) of the received signal. For signals with arbitrary shapes, reliable indications cannot be derived from simplified formulas, making the use of numerical tools imperative.

In the context of the considered ultrasonic positioning systems, simulations must encompass extensive spatial domains, spanning several cubic meters, and temporal windows extending over tens of milliseconds (see, for instance, [10,11,14]). A robust numerical instrument, the academic Field II software [24,25], has demonstrated its suitability for analyzing ultrasound positioning systems. It allows simulating any shape of transducers and ultrasonic signals for both transmission and reception. Moreover, Field II has the numerical features that allow simulating the large spatial regions and time windows required in the field of ultrasonic positioning. This tool is also able to take into account the attenuation properties of the propagation medium.

In a past study [26] using Field II, it was shown that a chirp emitted by a transducer with a certain aperture, and consequently its cross-correlation, undergoes a shape aberration that increases with a larger aperture. The larger the aperture, the narrower the useful emission cone within which the signal is sufficiently undeformed to estimate the time of arrival using the classical technique of identifying the lag of the global peak of the cross-correlation between the emitted and received signals. In other words, in the presence of signal aberration, in our case due to self-interference of the overall signal coming from a non-point-like transducer aperture, the desired lag no longer corresponds to any peak. Therefore, none of the known techniques (some of which are quite sophisticated) normally used for peak detection are applicable. In our case, the problem is quite different: finding the correct lag even without having the lag-peak correspondence available.

The conclusions drawn were that there is a practical limit to the maximum aperture of emitting transducers that can be used in an ultrasonic localization system, and the aperture is smaller as the frequency of the emitted signal increases. Unfortunately, the power of the emitted signal is also proportional to the effective surface area of the transducer, so having a small aperture means limiting the power of the signal and thus limiting the available signal-to-noise ratio (SNR).

The present work is a continuation of a series of studies focused on the acoustic aspects involved in ranging and localization based on ultrasonic signals. In particular, the numerical simulation of the acoustic field produced by transducers has been introduced for the first time to assess the acoustic field at every point in space and at every instant, in relation to the specific shape of the emitted signal. Thanks to this approach, certain purely acoustic aspects, crucial for the correct dimensioning of any ultrasonic ranging and positioning system, have been brought to the attention of the scientific community.

In particular, in a first article [26], we introduced the acoustic simulation tool Field II in the MATLAB environment, applied for the first time to ultrasonic transducers in air for ranging and positioning. In that work, we demonstrated how the signal propagating in a spatial region undergoes aberration, which becomes more severe as the angle between the transducer’s emission axis and the receiver (or sensor) increases, and as the diameter of the transducer’s emission surface increases.

It also emerged that the aberration, combined with the classical technique of acoustic signal time-of-flight detection, leads to unacceptable errors for transducers with apertures beyond a certain threshold. The need to use relatively small apertures consequently imposes significant limitations on the available signal power, which linearly depends on the transducer’s surface extension, and on the signal-to-noise ratio, crucial for the operability of positioning systems in real environments.

In a second work [17] building on the results from [26], it was shown that the Field II tool allows for estimating the effectiveness of a specific ranging technique in the presence of acoustic noise.

In a third study [27] utilizing Field II, a novel ranging method based on the differential attenuation of the acoustic signal at different frequencies was introduced. The technique was evaluated for its advantages and disadvantages and validated through simulations.

This work builds upon the findings in [26] to introduce a novel technique aimed at addressing aberrations in ultrasonic signals during their propagation through air. These aberrations arise from out-of-the-emission-axis auto-interference caused by the non-point-like emission aperture.

According to the principles of acoustics, the conventional solution to the signal aberration problem involved reducing the emission aperture size to approximate a point-like source. However, adopting this approach significantly diminishes the power of the emitted acoustic signal and, consequently, the available signal-to-noise ratio (SNR) at the receiver, all under the same boundary conditions (distance, environmental noise level, etc.). In contrast, the proposed technique enables the compensation of aberrations in transducers without any size reduction, resulting in improved ranging accuracy, higher output power, and, consequently, a higher SNR. This work provides a comprehensive and detailed description of the proposed technique and validates its effectiveness through experimentation using the Field II acoustic simulator. Furthermore, the technique is evaluated in the presence of varying levels of acoustic noise and for different values of an internal parameter.

This paper is structured as follows. Section 2 briefly introduces the simulation tool and setup, while Section 3 focuses on the problem to be addressed. Section 4 presents the proposed technique and the simulation results. Section 5 concludes the paper.

## 2. Brief Introduction to Field II and Simulation Setup

This section provides a concise overview of the operating principle of the Field II simulator [25] and offers a detailed description of the simulation setup. The simulator utilizes spatial impulse responses to model the ultrasound field for pulsed and continuous wave scenarios, employing linear systems theory [28,29,30]. The spatial impulse response describes the ultrasound field emitted at a specific spatial point over time when the transducer is excited by a Dirac delta function. The overall field generated by any excitation is determined by convolving the spatial impulse response with the excitation function. The technique divides the transducer surface into small rectangles, treating each as a rectangular piston with a known impulsive response [31]. The emitted spherical waves from these elements are then combined to calculate the impulsive responses at each desired field point. This approach allows for flexibility in considering various excitations while maintaining computational efficiency.

In the following simulations, the objective is to investigate the acoustic field and assess the efficacy of the proposed ranging technique within a standard 4 m × 4 m × 3 m room [32]. Specifically, the simulation results will be carried out for a grid of points forming a vertical section (refer to Section A in the room volume, see Figure 1 in [26]) and a horizontal section positioned midway between the floor and ceiling (refer to Section B in the room volume, Figure 1 in [26]). The transducer, a circular planar one, is centrally positioned on the ceiling at coordinates *x* = 0, *y* = 0, and *z* = 0, emitting downward toward the room floor.

The transducer operates in air, assuming a linearized air absorption model (slope 39.3 dB/m·MHz, constant term −0.262 dB/m, approximately 0.917 dB/m @ 20 kHz, and 1.703 dB/m @ 50 kHz) around 40 kHz. This corresponds to a transducer central frequency of 40 kHz under conditions of 20 °C temperature, 1 atm pressure, and 55% relative humidity [33,34].

The signals emitted and received at various points in the space are influenced by the shape and size of the transducer’s emission surface (i.e., aperture *D*). For this study, a circular plane transducer was selected, possessing similar acoustic properties to widely used transducers in positioning applications, such as the Murata MA40S4S piezoelectric transducer (*D* = 9.9 mm) or the Pro-wave 328ST/R160 (*D* = 13.1 mm) [35,36].

Capacitive transducers, on the other hand (e.g., models 600 and 7000 SensComp Inc., Livonia, MI, USA, etc.), ensure a broader bandwidth but, structurally, have larger apertures ranging from 15 mm to 37 mm. In [26], it was demonstrated that an aperture larger than 8.5 mm does not guarantee the required spatial coverage for indoor positioning applications. Better results were achieved with an aperture as small as 6 mm. Therefore, achieving optimal spatial coverage necessitates a significant reduction in the aperture of these capacitive transducers. However, mechanically reducing the aperture is not well-suited to these transducers and substantially decreases their available acoustic power.

The emitting disk is segmented into a specific quantity of rectangles, specifically square elements measuring 0.125 mm by 0.125 mm in all subsequent simulations. The chosen transducer element size in this study represents a well-balanced compromise between solution accuracy and the computational resources utilized in the simulation, as shown in Figure 2 in [26].

The signal employed in the simulations is a linear chirp spanning a bandwidth of 30–50 kHz and lasting 5.12 milliseconds [11,32]. For simulation purposes, the signal was sampled at a rate of f_S_ = 1 MHz. The acoustic field was computed at various points in space throughout a time window aligning with the complete reception duration of the signal [31].

After completing the simulation, the temporal behavior of the acoustic pressure generated by the entire excitation signal was obtained for each considered point in space. This facilitated subsequent assessments and post-processing of the signal, such as calculating peak pressure and total signal strength at each point. Following that, an ideal receiver was assumed, linearly transducing the pressure signal into an electrical signal after suitable sampling and numerical quantization. This process allowed for the calculation of the cross-correlation array ***C***.

## 3. Signal Aberration

In [26], the dependence of the signal aberration with the transducer emitting surface area or aperture was shown. The simulations included acoustic diffractive phenomena and frequency-dependent absorption phenomena, as anticipated in the previous section.

Figure 3c in [26] showed the results of the estimate of the range obtained using the usual technique, based on the search for the position of the cross-correlation peak (τ_MAX_) [16,37]. As a result, for the first four aperture diameters (i.e., 25, 20, 16, and 13.1 mm), the lag of the cross-correlation peak did not correspond to the correct time of arrival (TOA), and the range estimation differed by more than 1.5 cm. It also should be noted that the magnitude of this ranging error is amplified in the subsequent process of spatial coordinate estimation [11].

Further, the impact of using the larger aperture diameter, i.e., *D* = 25 mm, and the classic ranging technique based on global cross-correlation peak detection in a typical room was illustrated. With this transducer aperture, there was a significant signal shape aberration. However, on the other hand, as expected, the highest power level was achieved, resulting in a higher signal-to-noise ratio (SNR), which is highly desirable in noisy environments.

**Figure 1:**
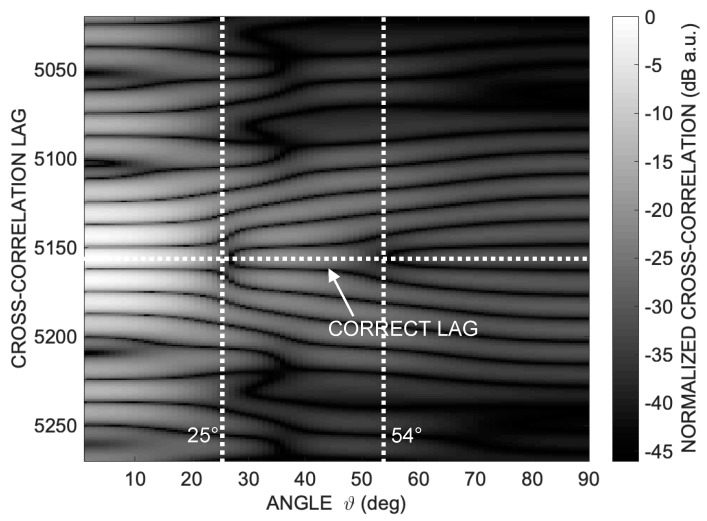
Log-compressed cross-correlation absolute values along a semicircular path at distance *R* = 1 m from the emitting transducer and transducer aperture *D* = 25 mm. It is possible to appreciate the progressive disappearance of a well-defined correlation peak as the angle *ϑ* increases and a double bifurcation at approximately 25° and 54°, which makes univocal identification of the TOA difficult.

In Figure 4a in [26], the ranging error was evaluated for *D* = 25 mm. It shows the ranging error along a rectangular vertical section (section of the room volume, Figure 1) of 3 m height and 4 m base passing through the center of the transducer, equal to the vertical section of the typical office room taken as a reference in some positioning works [32,38], while Figure 7b in [26] shows the behavior of the ranging error on a horizontal section of 4 m × 4 m at z = 1.5 m, or halfway between the ground and the ceiling. In both the figures, the grid pitch is 5 cm in the *x* and *y* directions.

In this figure, as discussed in [26], it is possible to clearly recognize the low-error areas, where the ranging errors are lower than about 3.09 mm. Such ranging error level is due to the numerical approximations and the sampling frequency chosen for the simulation. The simulation results therefore demonstrated that when using the aperture *D* = 25 mm, it is not possible to cover the whole room, which is certainly of interest for three-dimensional indoor positioning systems.

## 4. Signal Aberration Correction Technique and Numerical Results

As mentioned earlier, it is highly advantageous to use a transducer with the largest possible emitting surface to achieve high signal intensity and a high signal-to-noise ratio (SNR).

For instance, in Figure 3a in [26], it can be observed that the acoustic pressure level increases significantly when transitioning from *D* = 6 mm, which ensures excellent coverage of the entire volume of interest, to *D* = 25 mm, resulting in a gain of 20 dB on the axis and approximately 15 dB at 90°. However, this larger aperture suffers from substantial ranging errors just outside its emission semi-cone, spanning about 20°.

Therefore, there is a need to develop a new technique that, while exploiting the benefits of high emitted power and SNR with wide apertures, can extend the spatial region where the ranging error is sufficiently low for the positioning purpose to the entire volume of interest.

To achieve this goal, it is insightful to examine the cross-correlation along the same circular path depicted in Figure 1 in [26], namely along a quarter of a circumference within a plane passing through the transducer’s emission axis with a radius of *R* = 1 m. Figure 1 illustrates the absolute values of cross-correlation amplitudes in dB grayscale, plotted against lag and angle *ϑ*, for the considered aperture *D* = 25 mm.

While a well-recognizable cross-correlation peak (white pixels) is evident for *ϑ* less than 20°, there is a gradual disappearance of a well-defined correlation peak as *ϑ* increases. There is a double bifurcation at approximately 25° and 54°, complicating the unambiguous identification of the time of arrival (TOA).

**Figure 2:**
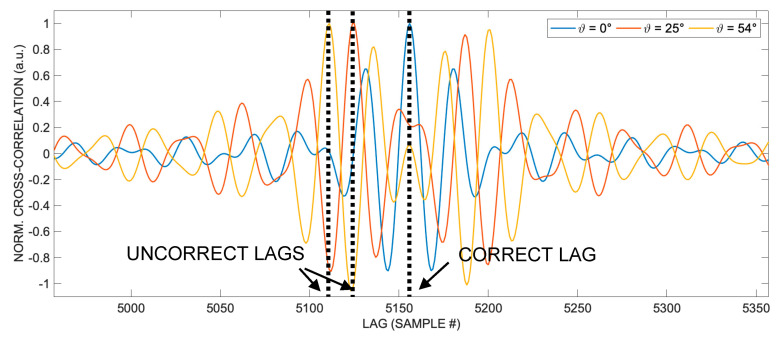
Cross-correlations along a semicircular path at distance *R* = 1 m from the emitting transducer for the transducer aperture *D* = 25 mm. It is possible to see that the single unique and well-recognizable peak of the cross-correlation for *ϑ* = 0° is no longer present at the 25° and 54° angles. The relative shape deformation with respect to the increasing angle is remarkable and represents a challenge for the correct TOA lag estimation. The cross-correlation values are normalized to their maximum values.

Figure 2 displays the cross-correlations along a semicircular path at a distance *R* = 1 m from the emitting transducer with aperture *D* = 25 mm at angles out-of-the-axis 0°, 25°, and 54°, respectively. In particular, three correlations at 0°, 25°, and 54°, respectively (which are three columns, represented linearly, of the matrix representing the log-compressed image of Figure 1) are shown. The cross-correlation values are normalized to their maximum values. It is possible to appreciate the progressive disappearance of a well-defined single correlation peak as the angle *ϑ* increases and the appearance of double peaks at the angles of approximately 25° and 54°, in the vicinity of which it is difficult to unambiguously identify the TOA.

Since the main cause of the increasing error with the angle *ϑ* from which the transducer is viewed is the self-interference of the acoustic signal from different regions of the same emitting surface of the transducer, which alters the signal shape at the receiver; the underlying idea of the proposed technique is to employ a more sophisticated approach than simple detection of the global peak of the cross-correlation to identify the lag corresponding to the correct time of arrival (TOA).

Examining the trend of the cross-correlation in Figure 1 provides a crucial observation. Indeed, it can be observed that at angles around 25° and 52°, the cross-correlation exhibits evident distortion and even a bifurcation in its shape, while elsewhere it remains relatively regular.

These bifurcations underlie the errors in Figure 3c in [26]. More specifically, it is noted that each cross-correlation, despite distorting and losing its unique peak at the correct TOA lag, remains substantially symmetrical around that point, as shown in Figure 2.

Furthermore, considering the points of the cross-correlation falling within a certain amplitude range, for example, with a value exceeding a certain percent of the global peak, thanks to the symmetry of the waveform, it is possible to estimate the center of this symmetry as the weighted average of the lag for each point, with good approximation. In other words, taking the set of points with values comparable to the global maximum (for example, in the range of 100–40% of the peak value) of the aberrated cross-correlation, we observe that its weighted average has a lag value very similar to that of the global maximum of the undistorted cross-correlation. Hence, the weighted average lag corresponds with good approximation to the correct lag, which in turn is proportional to the sought-after TOA.

**Figure 3:**
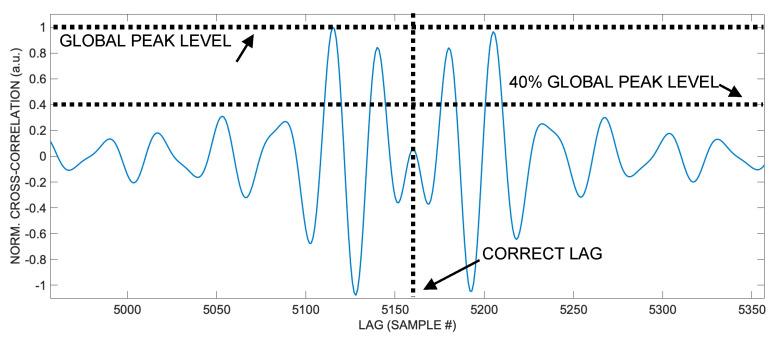
Aberrated cross-correlation shape at *ϑ* = 54°. Considering cross-correlation values higher than, for example, 40% of the global peak and calculating their weighted average lag can yield a good approximation of the correct lag, thanks to the intrinsic symmetry of the cross-correlation.

So, once the signal is received, to estimate the lag corresponding to the time of arrival (TOA), it is sufficient to calculate the cross-correlation, take all the points with a value exceeding a selected fraction of the global peak value, and compute the weighted average of their lags (see Figure 3).

**Figure 4:**
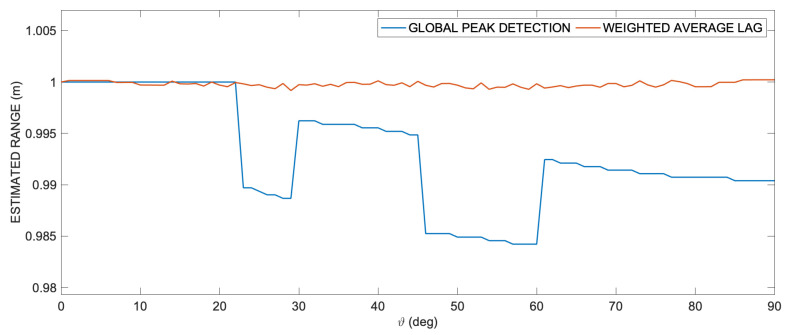
Estimated range comparison between direct detection of the position of the cross-correlation absolute peak and mean lag estimation of the set of the cross-correlation values down to the 40% of the peak value. It is important to notice that the lag of the cross-correlation peak does not allow to estimate the correct range for angles higher than approximately 20°, while the mean lag estimation yields a good approximation of the correct range at any angle.

The following simulations illustrate the results of the procedure described so far. In Figure 4, we compare the ranging results along an arbitrary path—specifically, the semicircumference with a radius of 1 m centered at the transducer—using both the conventional technique of cross-correlation global peak detection and the proposed method involving the weighted average of lags. As clearly evident, the effect of shape aberration is corrected, and it is possible to calculate the correct lag, thanks to the almost-symmetry of the aberrated cross-correlation.

The ranging we have seen is typically performed within the framework of the sensor position estimation process. Many ultrasound positioning systems (see, for example, [11]) use multiple range measurements to geometrically calculate the absolute position of one or more sensors within their reference system. The minimum number of required measurements is three, but some redundancy is employed to cope with possible ranging errors. In such systems, each measurement is performed separately; each transducer emits its signal at different times without overlap with others, or multiple transducers emit simultaneously, but each uses a coded signal to be strictly orthogonal to others. Additionally, the acoustic signal has limited duration (chirp burst of a few milliseconds), and the next burst is emitted after waiting sufficient time to make echoes in the room negligible. Therefore, the presence of more than one signal at a time in the room must be considered excluded.

However, in real applications, it is not always possible to correctly perform all the required range measurements. Indeed, a necessary condition for accurate ranging is the presence of a direct path free from the emitting transducer to the receiver, meaning the presence of a line of sight (LOS). If an obstacle partially obstructs the direct path, the measurement may not be possible at all or may be distorted. In such cases, the acoustic signal is still received by the sensor, but it has actually traveled a longer path than a straight line, bypassing the obstacle. This latter case is the most insidious of all because it is impossible to recognize by analyzing the signal itself. If this incorrect range is used to calculate the sensor’s position, the result will also be incorrect. In [11], the potential positioning error caused by incorrect ranging is avoided by subjecting the set of ranges required for localization to a geometric verification test. If the test is passed, the range measurements are accepted, and the position is calculated correctly; otherwise, at least one range measurement is incorrect. In such cases, the entire current set of ranges is discarded, and all distance measurements are performed again.

The problem of acoustic reflections remains to be addressed, together with the effects of ambient acoustic noise.

Acoustic reflection involves the receiver of the position sensor receiving a delayed replica of the original signal. The replica is generated when the acoustic wave reflects off an obstacle. The amplitude and shape of the reflected signal naturally depend on the physical properties of the reflector. The worst-case scenario is represented by a reflector consisting of a flat and perfectly reflective surface.

In such a case, the reflected signal can have the same amplitude as the direct signal. The only difference between the two is that the reflected signal arrives with a certain delay due to the difference in length between the path along the line of sight (LOS) and the indirect path, which, by definition, is always longer.

If the two paths, the direct and the reflection, differ by several centimeters or meters, then the two acoustic signals do not interfere with each other, and it is sufficient to consider the first signal that reaches the receiver. However, the situation becomes more complex if the two paths differ so little that the two signals significantly interfere with each other, creating an additional aberration in the shape of the cross-correlation.

By using Field II, it is possible to assess the effect of the overlap between the direct and reflected signals on the ability of the proposed technique to estimate the correct distance as the difference in length between the two paths varies.

The following simulation results illustrate how the proposed technique behaves in the presence of acoustic reflections. In all the simulations that follow, we have considered the worst-case scenario where the reflection coefficient is equal to 1, meaning the reflection with maximum amplitude equal to the direct signal. The transducer, the signal used, and other physical parameters remain the same as in the previous simulations.

**Figure 5:**
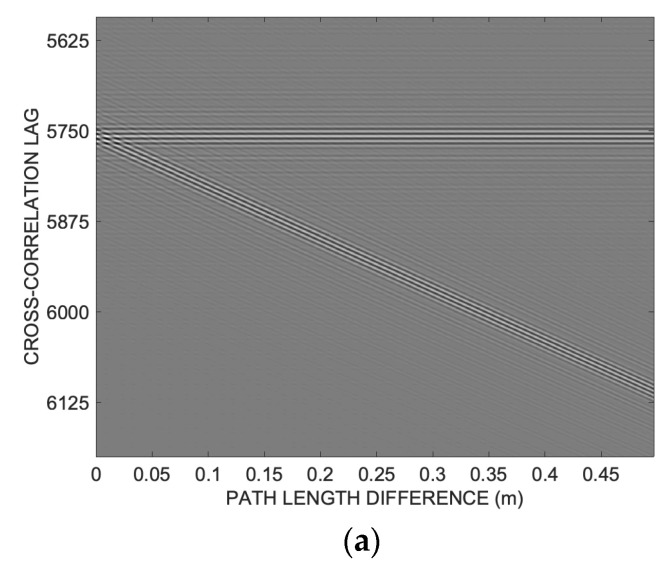

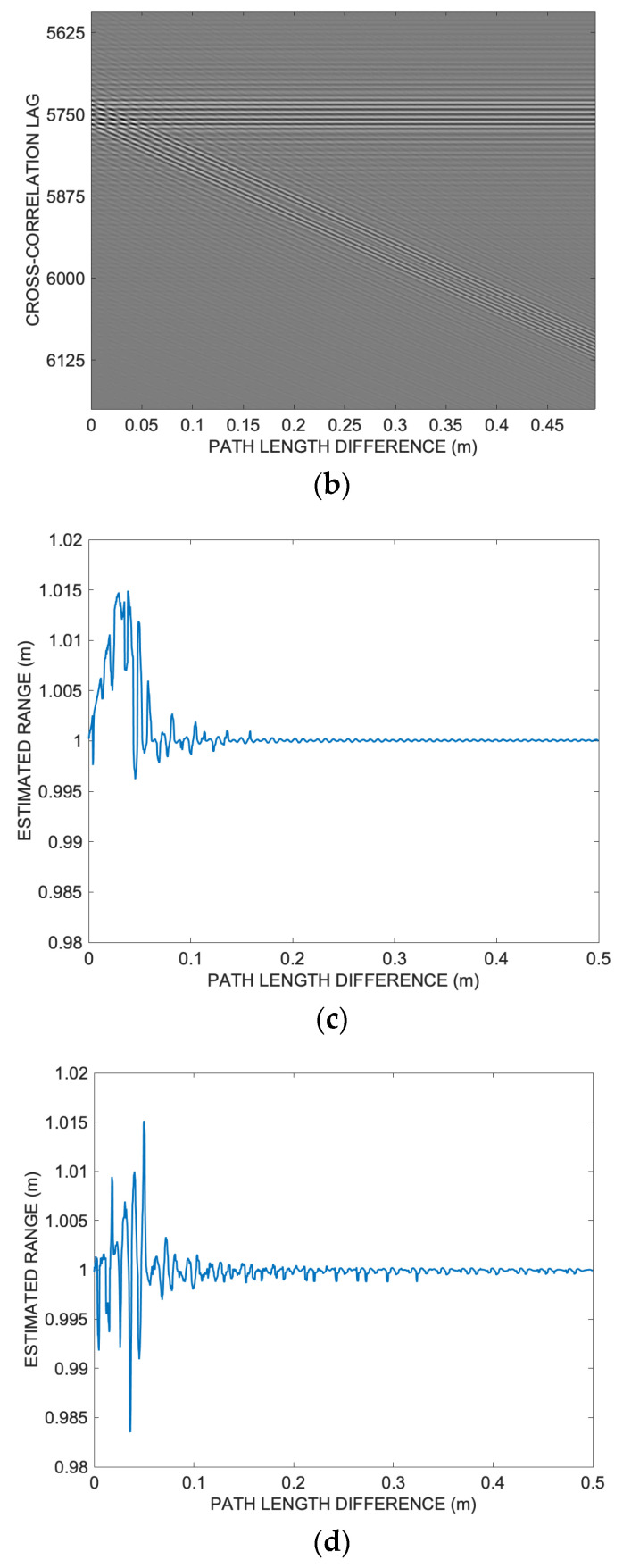
Effects of acoustic reflection: (**a**) shape of the cross-correlation between the received signal and the reference signal, varying the difference in length of the traveled path from the acoustic signal related to the line of sight (LOS) and the reflected path. The receiver is positioned at a distance *R* = 1 m from the transducer and observes it at an angle of 0°, i.e., it is on the transducer emission axis. Smaller cross-correlation lags correspond to a shorter distance from the transducer; (**b**) the receiver is positioned at a distance *R* = 1 m from the transducer and observes it at an angle of 54°; (**c**,**d**) ranging obtained using the proposed technique for the two signals in (**a**,**b**), respectively. Starting from a path length difference greater than the width of the peak train of the cross-correlation (here about 150 lags), it is possible to easily distinguish between the direct and reflected signals. For path length differences larger than approximately 5–6 cm, the range estimation is sufficiently accurate, differing by less than 2–3 mm from 1 m.

Figure 5a depicts the shape of the cross-correlation between the received signal and the reference signal, varying the difference in length of the traveled path from the acoustic signal related to the line of sight (LOS) and the reflected path. The receiver is positioned at a distance *R* = 1 m from the transducer and observes it at an angle of 0°, i.e., it is on the transducer emission axis. Smaller cross-correlation lags correspond to a shorter distance from the transducer. In Figure 5c, the ranging obtained using the proposed technique is shown. Starting from a path length difference greater than the width of the cross-correlation peak train (here about 150 lags), it is possible to easily distinguish between the direct and reflected signals. For path length differences larger than approximately 5–6 cm, the range estimation is sufficiently accurate, differing by less than 2–3 mm from 1 m.

In Figure 5b,d, the scenario is illustrated for an off-axis angle of 54°. In this case as well, for path length differences greater than about 5–6 cm, the range estimation differs by less than 2–3 mm from the correct one. As shown, for path length differences below 5–6 cm, the ranging error becomes significant, and the performed ranging is not useful for subsequent positioning computations.

This occurs when the path between the emission transducer and the sensor passes within approximately 5–6 cm of a reflective surface. Careful placement of emitters typically avoids many situations of this kind; however, there are rare cases where reflection cannot be avoided, representing a limitation for positioning systems with this type of architecture.

The following simulation results show how the proposed technique behaves in the presence of ambient noise.

**Figure 6:**
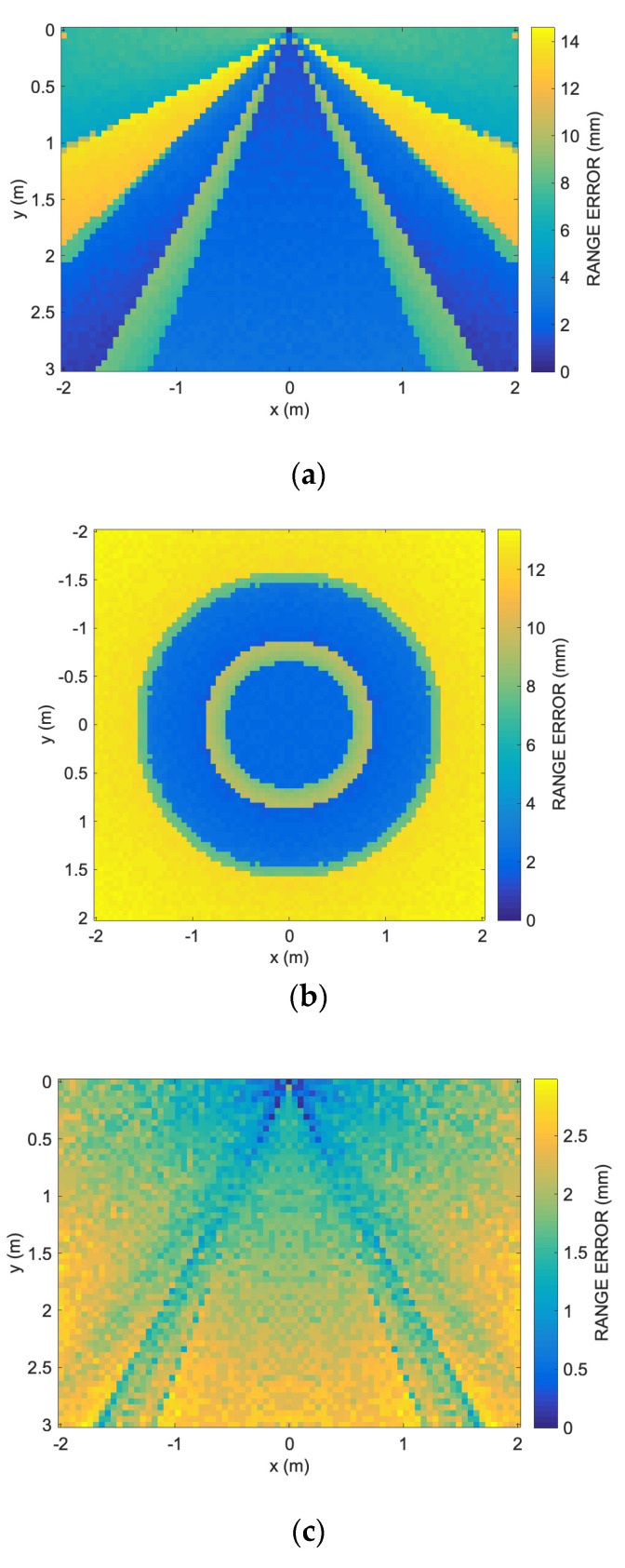

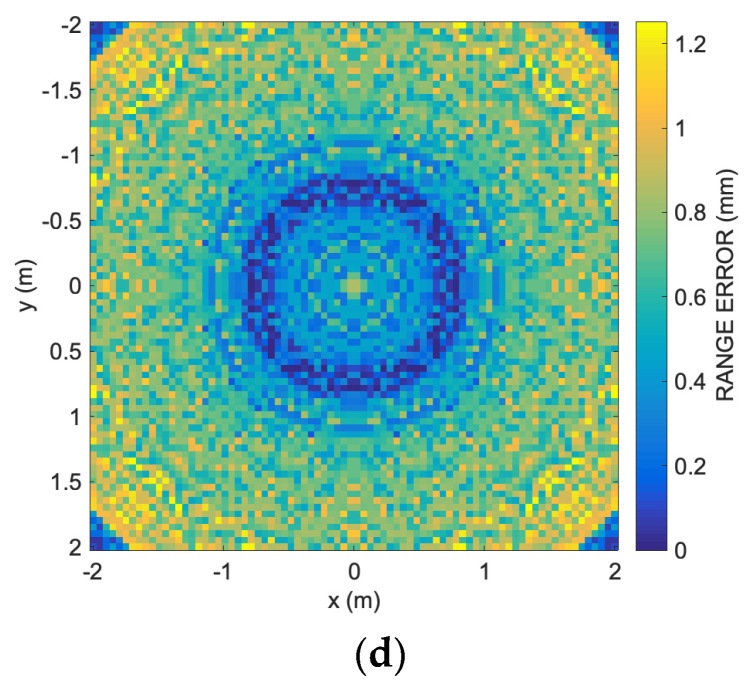
Comparison of the numerical results for range error using the transducer aperture (*D* = 25 mm; horizontal and vertical step = 0.05 m), with the global peak detection (**a**,**b**) and the weighted average lag techniques (**c**,**d**) for a vertical 4 m × 3 m room section (**a**,**c**) and a horizontal 4 m × 4 m room section at *z* = 1.5 m (**b**,**d**); in (**c**) the cone of minimum ranging error in the vicinity of the transducer axis in the half-space in front of the transducer has almost disappeared comparing with (**a**); in (**d**) the disk error pattern present in (**b**) is absent. The absolute error value is less than 3.0 mm in (**c**,**d**). Subfigures (**a**,**b**) are reproduced from [26] with permission of the authors.

Figure 6 displays the comparison of the numerical results for range error using the transducer aperture (*D* = 25 mm; horizontal and vertical step = 0.05 m) with the global peak detection and the weighted average lag techniques. In Figure 6a, the ranging errors generated by the global peak detection technique along the vertical 4 m × 3 m room section are displayed. Figure 6b shows the errors produced by the global peak detection technique along the horizontal 4 m × 4 m room section. Figure 6c shows the errors generated by the weighted average lag technique along the vertical 4 m × 3 m room section; it is possible to observe that the cone of minimum ranging error in the vicinity of the transducer axis in the half-space in front of the transducer has disappeared in comparison with (a). Figure 6d displays the error by the weighted average lag technique along the horizontal 4 m × 4 m room section. It is possible to see that the disk error pattern present in (b) is absent. Moreover, it is possible to observe that, using the proposed technique, the absolute error value is less than 3.0 mm everywhere.

White noise was added to the signal to obtain an SNR of 30 dB, which is the threshold usually considered for ultrasonic localization applications in environments such as offices and homes. The trend of the overall RMS error was then evaluated over the entire vertical room section for different threshold values, from 10% to 100% in steps of 10%; that is to say, the global peak value was multiplied by the sequence of weights from 0.1 to 1 in steps of 0.1.

**Figure 7:**
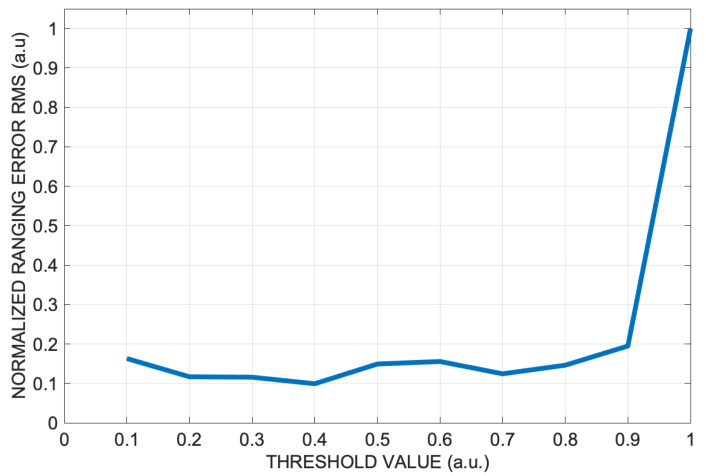
Normalized ranging RMS error along the vertical 4 m × 3 m room section versus the threshold values from 0.1 to 1 at step of 0.1.

In Figure 7, it is possible to see the trend of the normalized ranging RMS error as the threshold varies. It can be seen that the RMS error increases dramatically when the threshold value goes from 0.9 to 1. This is an expected effect since for the threshold value of 1, the weighted average lag technique is reduced to simple global peak detection. The simulation data shows that there is little difference using a threshold between 0.1 and 0.9; however, the value of 0.4 produces the global minimum, while the value 0.7 still gives a good result with slightly fewer calculations.

Once the value for the 0.4 threshold was selected as adequate, the simulations were then repeated at decreasing SNR levels, from 30 dB down to −20 dB in steps of 10 dB, this time only for the vertical section.

**Figure 8:**
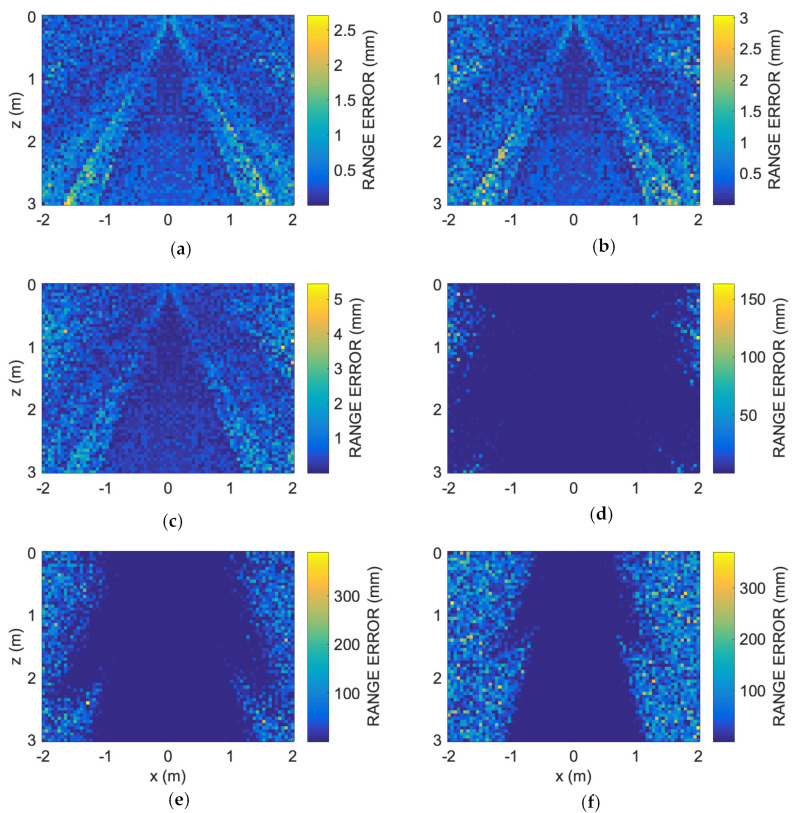
Numerical results at different SNR levels. Spatial error distribution along the room vertical section: (**a**) SNR = 30 dB, (**b**) SNR = 20 dB, (**c**) SNR = 10 dB, (**d**) SNR = 0 dB, (**e**) SNR = −10 dB, (**f**) SNR = −20 dB. Note the color bar error different ranges. For decreasing SNR from 30 dB down to 10 dB, the error is equal to or less than 5 mm. For lower SNR, error rapidly increases up to more than 300 mm, mainly in the out-of-the-axis regions.

Figure 8 shows the range estimation error along the 4 m × 3 m vertical room section for decreasing SNR levels from 30 dB down to −20 dB.

**Figure 9:**
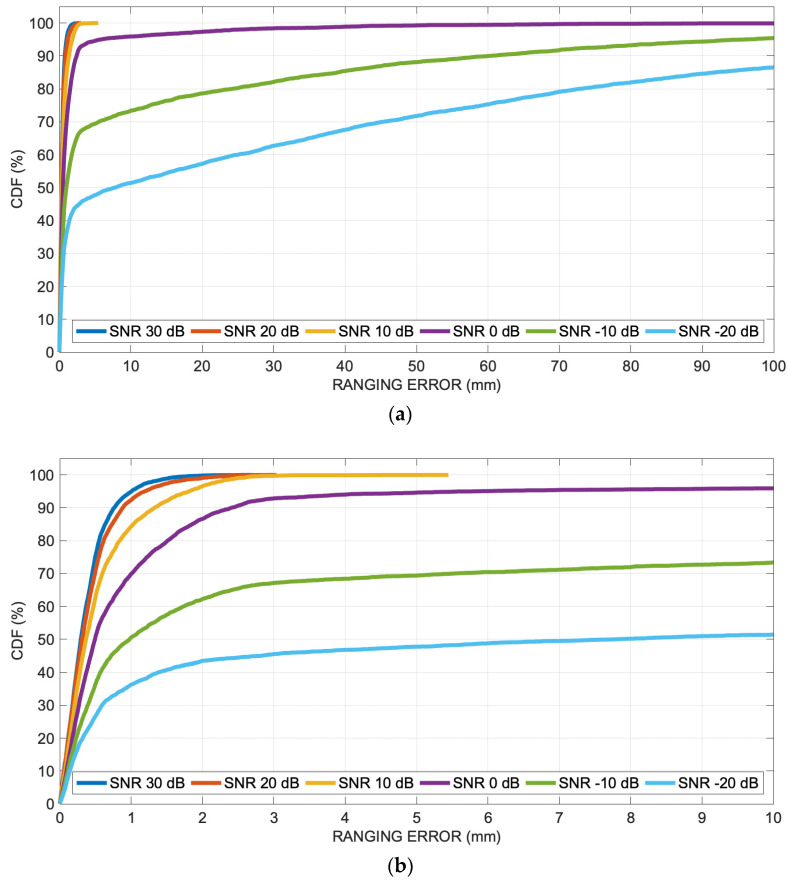
Numerical results at different SNR levels: (**a**) cumulative distribution function (percent of readings with error less than the value of a given abscissa) of the ranging error along the vertical room section for decreasing SNR levels from 30 dB down to −20 dB; (**b**) *x*-axis zoomed portion of the cumulative distribution function in the range 0–10 mm.

Figure 9a displays the overall ranging error at any grid point from Figure 8 as a cumulative distribution function (CDF) or cumulative error distribution, which is the percentage of readings with error less than the value of a given abscissa, in the range 0–100 mm. Figure 9b shows a zoomed portion of the CDF in the range 0–10 mm, in order to see the behavior details of the ranging mechanism for SNR levels from 30 dB down to −20 dB.

## 5. Conclusions

The article presents a method for correcting signal aberrations in ultrasonic indoor positioning. The challenges associated with signal aberrations in ultrasonic systems, particularly when the aperture of the transducer exceeds a certain limit, causing shape aberrations in the typical linear chirp signal, are addressed. These aberrations impact the cross-correlation technique, leading to errors in range estimation.

To overcome this challenge, the authors propose an alternative technique based on the inherent symmetry observed in the shape of the aberrated cross-correlation. The proposed technique involves calculating the weighted average of lags for points with cross-correlation values exceeding a certain threshold. This method aims to correct the errors introduced by shape aberrations and improve the accuracy of range estimation, allowing for the use of transducers with larger apertures.

The numerical simulations, conducted using the Field II acoustic simulation software, compare the classical cross-correlation technique with the proposed weighted average lag technique in a standard office room. The results demonstrate that the proposed technique enables accurate range estimation even in the presence of severe cross-correlation shape aberrations.

Furthermore, the ability of the proposed technique to cope with acoustical reflections has been evaluated. The technique accurately estimates the range even in the presence of acoustical reflections, except in rare cases where the direct and reflected paths differ by less than 5–6 cm.

Moreover, the technique is tested at different SNR levels, demonstrating that acceptable results can be obtained with SNR of at least 10 dB in the selected environment. Furthermore, the dependence of the range error with the threshold values was investigated with numerical simulations, finding that a good choice for the threshold is a value equal to 40% of the global peak of the cross-correlation, as a rule of thumb.

Based on the obtained results, the proposed technique allows for the use of transducers with larger apertures, leading to higher acoustic power and improved signal-to-noise ratio (SNR) and expanding the applicability of ultrasonic systems for indoor positioning and tracking.

## Data Availability

Data are contained within the article.
